# Discovery of novel, orally bioavailable, antileishmanial compounds using phenotypic screening

**DOI:** 10.1371/journal.pntd.0006157

**Published:** 2017-12-29

**Authors:** Diana Ortiz, W. Armand Guiguemde, Jared T. Hammill, Angela K. Carrillo, Yizhe Chen, Michele Connelly, Kayla Stalheim, Carolyn Elya, Alex Johnson, Jaeki Min, Anang Shelat, David C. Smithson, Lei Yang, Fangyi Zhu, R. Kiplin Guy, Scott M. Landfear

**Affiliations:** 1 Department of Molecular Microbiology & Immunology, Oregon Health & Science University, Portland, Oregon, United States of America; 2 Department of Chemical Biology and Theraputics, St. Jude Children’s Research Hospital, Memphis, Tennessee, United States of America; University of Texas at El Paso, UNITED STATES

## Abstract

Leishmaniasis is a parasitic infection that afflicts approximately 12 million people worldwide. There are several limitations to the approved drug therapies for leishmaniasis, including moderate to severe toxicity, growing drug resistance, and the need for extended dosing. Moreover, miltefosine is currently the only orally available drug therapy for this infection. We addressed the pressing need for new therapies by pursuing a two-step phenotypic screen to discover novel, potent, and orally bioavailable antileishmanials. First, we conducted a high-throughput screen (HTS) of roughly 600,000 small molecules for growth inhibition against the promastigote form of the parasite life cycle using the nucleic acid binding dye SYBR Green I. This screen identified approximately 2,700 compounds that inhibited growth by over 65% at a single point concentration of 10 μM. We next used this 2700 compound focused library to identify compounds that were highly potent against the disease-causing intra-macrophage amastigote form and exhibited limited toxicity toward the host macrophages. This two-step screening strategy uncovered nine unique chemical scaffolds within our collection, including two previously described antileishmanials. We further profiled two of the novel compounds for *in vitro* absorption, distribution, metabolism, excretion, and *in vivo* pharmacokinetics. Both compounds proved orally bioavailable, affording plasma exposures above the half-maximal effective concentration (EC_50_) concentration for at least 12 hours. Both compounds were efficacious when administered orally in a murine model of cutaneous leishmaniasis. One of the two compounds exerted potent activity against trypanosomes, which are kinetoplastid parasites related to *Leishmania* species. Therefore, this compound could help control multiple parasitic diseases. The promising pharmacokinetic profile and significant *in vivo* efficacy observed from our HTS hits highlight the utility of our two-step phenotypic screening strategy and strongly suggest that medicinal chemistry optimization of these newly identified scaffolds will lead to promising candidates for an orally available anti-parasitic drug.

## Author summary

Leishmaniasis, caused by the protozoa of the *Leishmania* species, represents a spectrum of diseases that afflicts roughly 12 million individuals worldwide. Current drug therapies for this parasitic disease are suboptimal because they are toxic, expensive, difficult to administer, and subject to drug resistance. In order to identify new and improved drug candidates, we screened a large library of small molecules for compounds that inhibit parasitic growth inside mammalian host macrophages, and have low toxicity toward the macrophages. We discovered two compounds that significantly impaired disease progression when administered orally in an animal model of cutaneous leishmaniasis. The promising pharmacokinetic and *in vivo* efficacy profile of the compounds make them attractive starting points for pharmaceutical development.

## Introduction

Leishmaniasis constitutes a spectrum of diseases that range in severity from self-healing to fatal. The disease can present as self-healing but potentially disfiguring cutaneous leishmaniasis [1]; metastatic and highly disfiguring mucocutaneous leishmaniasis [2]; or fatal visceral leishmaniasis [3], where the parasite targets internal organs such as the liver, spleen, and bone marrow. Different species and strains of *Leishmania* parasites cause these distinct pathologies. The severity of the disease also depends upon host factors such as immune status [4]. An estimated 12 million individuals are infected with leishmaniasis worldwide, with a widespread geographic range that spans from India to the Mediterranean countries, to North and South America [5]. All *Leishmania* species have a life cycle that includes motile promastigotes that reside in the gut of the sand fly vector and non-motile amastigotes that live in the phagolysosomal vesicles of mammalian host macrophages [5].

Despite the disease’s prevalence, the current antileishmanial drug therapies are inadequate [6]. Since the 1940s, standard therapies for leishmaniasis include pentavalent antimonials, such as sodium stibogluconate (Pentostam) and meglumine antimonate (Glucantime), which are administered daily over the course of 20–30 days. Both drugs are subject to widespread resistance and are highly toxic such that treatment alone can lead to mortality [7]. The diamidine pentamidine, which has similar disadvantages, has been another drug of choice to treat cutaneous leishmaniasis for several decades. Newer drugs include amphotericin B, especially in liposomal formulation (AmBisome), the aminoglycoside paromomycin, and the phospholipid miltefosine [8, 9], which received FDA approval in 2014. However, none of these drugs is even close to optimal. They all have moderate to high toxicity, need to be administered over multiple weeks, and suffer from increasing drug resistance. Only miltefosine, a known teratogen that is unsuitable for pregnant patients, can be administered orally [10]. Leishmaniasis has been characterized as ‘a major health problem, and there is no satisfactory treatment so far’ [6]. Hence there is an urgent need for novel therapies that are safe, potent, orally bioavailable, have a low cost of goods, and are effective against drug-resistant strains of *Leishmania* parasites. Although a major bottleneck in progress had been the paucity of lead compounds [11] that offer the potential of becoming new antileishmanial drugs, the situation has improved recently with the application of phenotypic screening and the associated identification of multiple lead series [12].

Phenotypic screens measure the effects of a compound on intact cells rather than an isolated target (i.e., biochemical enzymatic assay) [13, 14]. Active compounds generated from whole cell-based phenotypic screens generally offer favorable cell permeability and solubility that can facilitate compound development. One limitation with this approach is that the mechanism of action of new compounds is typically unknown. Nonetheless, phenotypic screens have the complementary advantage that they can identify compounds that act therapeutically against pathways that were previously not known to be critical for parasite viability [15].

Prior phenotypic screens have predominantly used the promastigote form of the parasite, which can be readily cultured *in vitro* but is not the disease-causing form of the parasite. This approach has the advantage of being able to accommodate large numbers of compounds, such as the 200,000-compound library that Sharlow and colleagues screened [16]. Investigators have also used axenic amastigotes [17, 18], which are more relevant to the disease but are nevertheless a host cell-free system that only imperfectly approximates intracellular amastigotes. Most scientists agree that assays that use intramacrophage amastigotes are the most physiologically relevant assays even though they offer lower throughput. Researchers have started employing a two-stage approach involving an initial screen of promastigotes or axenic amastigotes and a secondary step to confirm the hits by screening them against intracellular amastigotes [19–21]. This approach allows the screen to be carried out with a facile high throughput approach followed by a second, more stringent, test of the primary hits for efficacy against the disease-causing intra-macrophage parasites.

The advent of high-content microscopic approaches has enabled the direct screening of compounds against amastigotes growing inside cultured mammalian macrophages [22–26]. This method can eliminate compounds that act against promastigotes while leaving amastigotes unaffected. This method is also useful for identifying compounds that target amastigotes but not promastigotes. However, this assay is technically much more complicated to undertake than assays that use promastigotes or axenic amastigotes [19]. Although one can screen large libraries with sufficient time and effort, the screens published to date have all employed smaller libraries, such as the 26,500-compound library used in Siqueira-Neto et al.’s report [26], or the focused libraries of Medicines for Malaria Box [27], and the microbial extracts collection [28].

Although many of the hits identified in the above screens have not yet been advanced to testing in animal models of leishmaniasis [29, 30], some promising leads have been identified, and various organizations are currently conducting medicinal chemistry programs. For example, the Drugs for Neglected Diseases Initiative (DNDi) is subjecting several chemotypes such as the nitroimidazoles and oxaboroles [31] to both *in vitro* and *in vivo* evaluation as orally deliverable antileishmanials in mice and hamsters. Furthermore, the Genome Institute of the Novartis Research Foundation (GNF) has identified a selective inhibitor of the kinetoplastid proteasome, GNF6702, which is active against several parasite species [32]. In addition to these advances, there is substantial benefit to providing a continued robust pipeline of lead compounds for the development of safe, potent, and orally bioavailable antileishmanials that could considerably improve the current sub-optimal armamentarium for leishmaniasis.

In this paper we report a screen of roughly 600,000 compounds for growth inhibition of *L*. *mexicana* promastigotes from several libraries, namely the St. Jude Children’s Research Hospital Chemical Biology & Therapeutics (CBT) library [33] and the Tres Cantos Antimalarial Set [34]. Two top hits from this screen, compounds **4** and **5,** exhibited promising pharmacokinetic profiles that were substantially efficacious in a *L*. *mexicana* murine model of cutaneous leishmaniasis when delivered by oral gavage at a dose of 25–30 mg/kg over 10 days. Together these results suggest that compounds **4** and **5** are promising new starting points for the development of orally bioavailable antileishmanial drugs.

## Materials and methods

### Animal studies statement

Animal work was approved by the Oregon Health & Science Institutional Animal Care and Use Committee under protocol #IS00002639 under adherence to the Animal Welfare Act regulations and Public Health Service Policy for the Humane Care and Use of Laboratory Animals or by the St. Jude Children’s Research Hospital Institutional Animal Care and Use Committee under protocol #477 in compliance with the Animal Welfare Act and rules articulated by the Public Health Service Policy for the Humane Care and Use of Laboratory Animals.

### Composition of CBT library

The current CBT library consists of roughly 600,000 unique molecules purchased from a variety of commercial sources. The library breaks into four major sets: approved drugs (~1,100 compounds); other known bioactives (~2,500 compounds); focused sets directed at defined targets, including G protein coupled receptors, kinases, proteases, and phosphatases (~45,000 compounds); and the diversity collection, which is the largest component of this library. All samples in the CBT library were carefully chosen to provide a balanced, functionally diverse collection suitable for discovery of chemical matter active against a wide variety of targets and for phenotypic screening [35, 36]. In particular, the diversity subset has been designed using a maximally diverse cluster philosophy so that the population is made up of multiple clusters, each containing a series of related compounds, where the clusters are diverse with respect to one another.

First, commercially available compounds were filtered using a combination of physiochemical metrics to improve bioavailability, and functional group metrics to reduce the probability of non-specific or artifact effects. The former is guided by the correlation of physiochemical parameters with bioactivity, as opposed to oral availability [36]. The latter is guided by implementation of the Vertex ‘Rapid Elimination of Swill’ model [37–39], which utilizes a numeric scoring method with each functional group being assigned a score from –5 (always excluded) to 0 (never excluded) and allowing an aggregate score of –2 before elimination. Next, the filtered compound list was used to generate maximally diverse clusters. In order to do this, the compounds were reduced to core fragments (or ‘scaffolds) using the method of Bemis and Murcko [40], and the compound clusters were then prioritized for purchase based on the balance of cluster diversity from the existing library as assessed by Tanimoto similarity and the presence of a reasonable number of analogs within a cluster. From 5 to 20 compounds per cluster were required, with preference for clusters of more than 20 compounds, from which a maximum of only 20 representative compounds were purchased.

### Quality control for purchased compounds

All materials were purchased from commercial suppliers and used without further purification. All hits subjected to further study were repurchased and identity and purity were assessed by ultra-performance liquid chromatography (UPLC) using an H-class Waters Acquity system. Data were acquired using Masslynx v.4.1 and analyzed using the Openlynx software suite. The total flow rate was 1.0 mL/min and gradient program started at 90% A (0.1% formic acid in H_2_O) and was changed to 95% B (0.1% formic acid in acetonitrile) and then to 90% A. A full scan ranging from m/z 110 to 1000 in 0.2 s was used to acquire MS data. Compound identity was confirmed by low-resolution mass spectrometry and purity was assessed by ultraviolet spectroscopy and evaporative light scattering. All samples were required to exhibit > 85% purity.

### High-throughput assay for growth inhibition of promastigotes

Into each well of 384-well microplates (black polystyrene, clear bottom, tissue culture treated, Corning), 15 μl of medium (DME-L [41] plus 100 μM xanthine and 10% heat inactivated fetal calf serum) was dispensed with a liquid dispenser (Matrix Wellmate, Thermo Scientific). Stock compounds, dissolved in DMSO at a fixed concentration of 10 mM, were pin-transferred (V&P Scientific) into the microplate to the desired final concentration using an automated robot arm. To each well of the plates, 15 μl of *L*. *mexicana* promastigotes (strain MNYZ/BZ/62/M379, 2 x 10^6^/mL) was added with the Wellmate dispenser. Microplates were incubated (Liconic) at 28°C and 5% CO_2_ for 72 h. After incubation, 10 μl of lysis/dye solution (5X SYBR Green I, 5% Triton X-100 in PBS) was added to each well. Plates were shaken at 1000 rpm, incubated at room temperature for 20 min, and fluorescence signal measured (excitation 485 nm, emission 535 nm) with the Envision plate reader (PerkinElmer).

### Assays for growth inhibition of *Leishmania* intracellular amastigotes and of *T*. *brucei* bloodstream forms

*L*. *mexicana* (MNYZ/BZ/62/M379) or *L*. *donovani* (LdBob strain) [42] parasites expressing the *Renilla* luciferase gene from a rRNA gene locus were used to infect J774A.1 macrophages. Growth of intracellular amastigotes was measured using a luminescence assay, as detailed previously [43]. The growth inhibitor activities of compounds were tested against bloodstream form Lister 427 *T*. *brucei* in 96-well plates containing 1 X 10^5^ parasites per well in 0.2 ml HMI-11 medium (Gibco/Thermo Fisher) [44]. Compounds (2 μl volumes in DMSO) were added to the parasites using serial 3-fold dilutions to cover a range of concentrations from about 10 μM to 1 nM. After 48 h incubation at 37°C under a humidified 5% CO_2_ atmosphere, 10 μl of 10% Triton X-100 and 100 X stock SYBR Green I (Sigma-Aldrich) in PBS was added and florescence measured (excitation 497 nm; emission 520 nm) after 1 h incubation in the dark using a Spectra Max Gemini XPS fluorimeter (Molecular Devices). Data were log transformed and EC_50_ values were determined using GraphPad Prism 6 (GraphPad Software). In the absence of growth inhibiting compounds, the parasites grew from an initial density of 5 X 10^5^ cells/mL to ~3 X 10^6^ cells/mL.

### Assays for growth inhibition of *Trypanosoma cruzi* epimastigotes

*Trypanosoma cruzi* CL Brener epimastigotes were obtained from Dr. Fred Buckner of the Department of Medicine at the University of Washington. *T*. *cruzi* epimastigotes were grown in liver infusion tryptose medium and seeded in a 96-well plate at 10^5^ epimastigotes per well in 100 μl medium. For each well, 1 μl of compound in DMSO at 100X the desired final concentration was added. Epimastigotes were exposed to a range of compound concentrations from 10 μM to 1 nM to determine EC_50_. Plates were incubated at 26°C for 72 h, then 10 μl 50X SYBR Green in 1% Triton X-100 was added to each well followed by incubation with shaking at room temperature for 30 minutes. Fluorescence was read (excitation 485nm, emission 535nm) with the Victor^2^ multiplate reader (PerkinElmer). All data processing and visualization were performed using GraphPad Prism 6 software.

### Assays for ADME and pharmacokinetics

Methods for determination of liver microsomal stability, solubility, permeability of artificial membranes, Caco-2 cell permeability, stability in simulated gastric fluid, binding to mouse serum proteins, and *in vivo* pharmacokinetic studies are reported in Supporting Information.

### Mammalian cytotoxicity assay

The BJ cell line was purchased from the American Type Culture Collection (ATCC, Manassas, VA) and cultured according to recommendations. Cell culture media were purchased from ATCC. Cells were routinely tested for mycoplasma contamination using the MycoAlert Mycoplasma Detection Kit (Lonza). Cells were grown to 80% confluence, collected, and plated in 25 μL of medium per well in 384-well plates (Costar 3712). Compounds were diluted as described above and transferred to cells using a pin tool (V&P Scientific) equipped with FP1S50 pins resulting in final compound concentrations of 25 μM, and the plates incubated for 72 h at 37°C in 5% CO_2_. CellTiter-Glo (Promega) detection reagent was added following the manufacturer’s instructions, and luminescence was measured using an EnVision (PerkinElmer) plate reader. Data were log transformed and EC_50_ values were determined using GraphPad Prism 6 (GraphPad Software).

Cytotoxicity of compounds to J774.A1 macrophages was determined by dose-response curves as described previously [43]. In the absence of growth inhibitors or DMSO, the macrophages increased in number ~6-fold over 96 h in Minimum Essential Medium, employed for both macrophage infections and the toxicity assays.

### 10-day toxicity study

Drug doses were chosen based on pilot toxicology and pharmacokinetic studies. Female BALB/C mice (compound **4**) or C57BL6 (compound **5**) of 17–21 grams were purchased from Charles River Laboratories (Wilmington, MA). Food and water were provided *ad libitum*. Two mice were used as control and another 5 mice were dosed daily via oral gavage (25 mg/kg with compound **4** and 50 mg/kg with compound **5**). Every day blood was collected by retro-orbital bleed from one animal from the treatment group for pharmacokinetics. Because compound **5** induced seizures when delivered at 50 mg/kg, blood glucose was simultaneously measured with a glucose meter (Alpha track) to determine whether reduced sugar levels could be a cause of this toxicity. Each mouse received two blood collections and glucose measurements over the 10-day course of treatment.

### Efficacy studies using murine model of cutaneous leishmaniasis

Female BALB/c mice (~20 g) were injected in one hind footpad with 1 x 10^6^ stationary phase promastigotes suspended in 25 μl of phosphate buffered saline (PBS). Four weeks after infection, when a small cutaneous lesion was visible in the injected footpad, cohorts of five mice were treated with either compound or vehicle alone (90 μl), delivered daily for 10 consecutive days by oral gavage using a 20-gauge x 30 mm disposable plastic feeding needle. Vehicle consisted of 10/10/40/39 mixture of ethanol/(PG)/PEG400/PBS plus 1% (weight/volume) 2HβCD (PG is propylene glycol, PEG is polyethylene glycol, 2HβCD is 2-hydroxy-β-cyclodextran). The daily dose for each compound was: compound **4**, 25 mg/kg; compound **5**, 30 mg/kg; miltefosine, 20 mg/kg. The width of the footpad (top to bottom) was measured with calipers before injection of parasites (day 0) and weekly from weeks 4–12. The width of the uninfected contralateral footpad was also measured each week, and its width was subtracted from that of the infected footpad to determine lesion size.

### Research involving animals

All research involving animals was carried out with the approval of the Institutional Animal Care and Use Committee of either St. Jude Children’s Research Hospital or the Oregon Health & Sciences University. The study was conducted adhering to the guidelines for animal husbandry of each institution.

## Results

### Identification of antileishmanial hits

**Fig 1:**
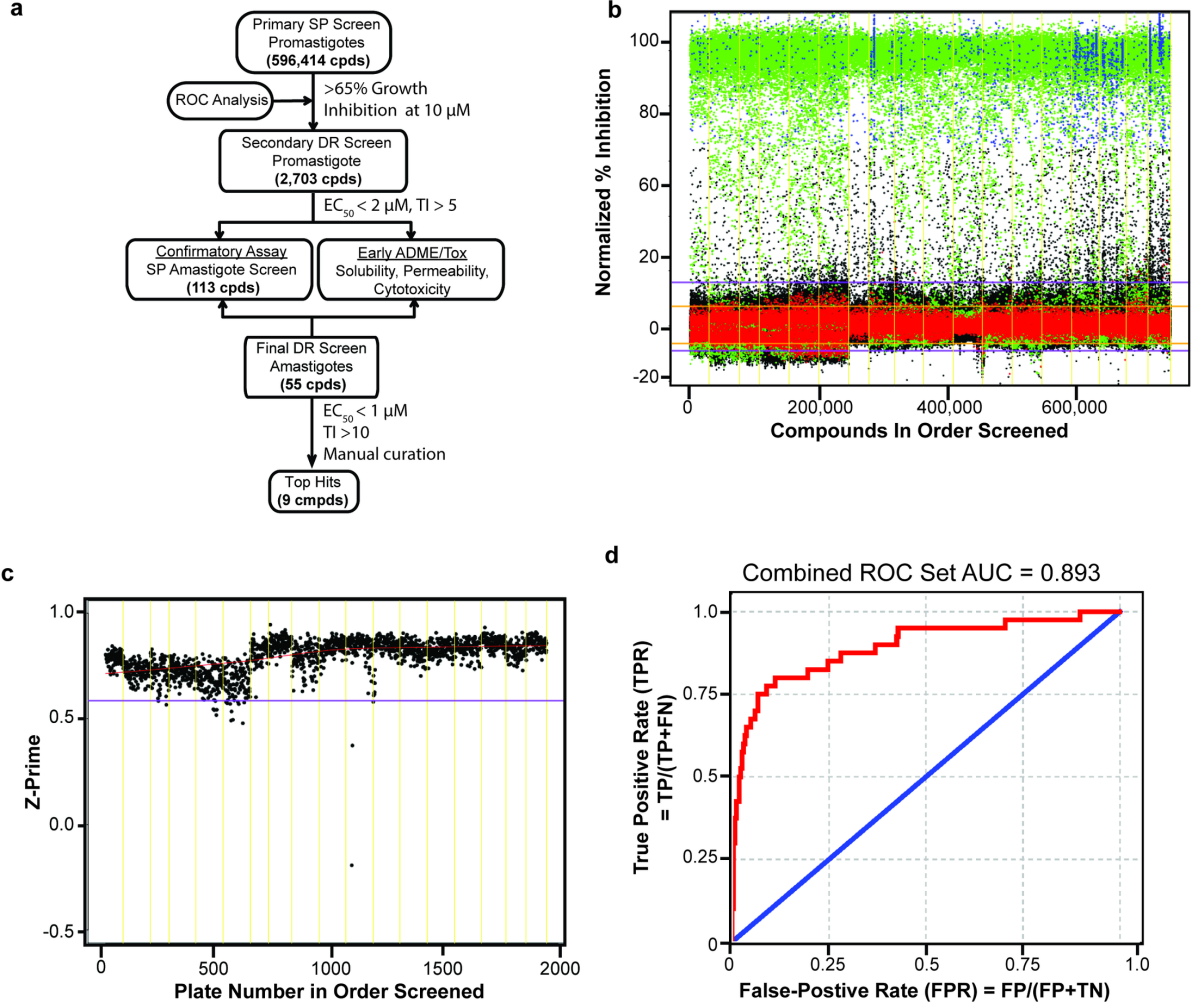
Screening flow chart and high throughput primary screen of promastigotes. **a.** Schematic of the high-throughput screening workflow. SP refers to single point, or single concentration, and DR represents dose-response. The cutoff value of 65% inhibition in the first step was chosen based in ROC analysis, the cutoff of 2 μM and TI > 5 in step 2 was somewhat arbitrary but produced a reasonable number of hits for subsequent analysis, and the cutoff of 1 μM and TI > 10 for the final step is consistent with recommendations for lead identification for leishmaniasis [12]. **b.** Scatter plot of primary screen data shown as normalized percent growth inhibition. Each dot represents the activity of one compound. Negative controls (DMSO treated) are in red, positive controls (pentamidine treated) are in green, test compounds are in blue (hits) or black (non-hits). The orange and purple horizontal lines indicate the 95% and 99% quantiles of activity respectively. **c.** Primary screen quality control: Z-prime value per assay plate screened, lower outlier bound (in purple), yellow lines separate screen runs. **d.** Receiver operating characteristic (ROC) curve (red); the combined ROC set AUC is 0.893. The blue line represents an AUC of 0.5 that is indicative of an assay with random results. TP is true positive, FP is false positive, FN is false negative, and TN is true negative.

A summary of the HTS workflow and quality control data is shown in Fig 1. The initial promastigote screen was performed with the St. Jude Chemical Biology & Therapeutics (CBT) library consisting of 596,414 compounds. Library compounds were filtered by several computational methods [35, 36] to remove those likely to have undesirable physical or biological properties and biased towards oral bioavailability. In this way we focused the collection on compounds most likely to be effective in cellular models of activity, without structural features that would pose a challenge to drug development [33]. In the primary screen, compounds were applied to promastigotes of *L*. *mexicana* at a fixed concentration of 10 μM, and parasite proliferation was monitored, following a 72 h incubation, by quantifying total DNA content after lysis using the nucleic acid binding dye SYBR Green I [45].

The raw data for the HTS campaign are summarized in Fig 1B as a scatterplot of normalized percent growth inhibition relative to the control drug pentamidine, which gives 100% inhibition of proliferation under these conditions. The scatterplot demonstrated ample signal separation between the positive (green) and negative (red) controls throughout the HTS campaign and a well-defined activity distribution of test compounds (blue and black). The fidelity and quality of the HTS assay were assessed using two metrics: Z-prime and EC_50_ of the control (pentamidine) that were calculated for each screening plate. The entire screen produced a median Z-prime value of 0.81 (interquartile range: 0.75–0.85, Fig 1C) and a consistent EC_50_ value of pentamidine (median 2.3 μM, interquartile range: 1.7–3.1) indicating the assay was consistent throughout the screen.

The assay’s discriminatory power was assessed using Receiver Operator Characteristic (ROC) analysis [46] as described [33]. This method helped define an optimal cutoff for designating primary hits by balancing the likelihood of a true positive with acquiring a reasonable total number of hits. Briefly, compounds were stochastically selected from the HTS screening set to sample the primary assay results according to the distribution of observed activities (ranging from 0 to 100% activity). The selected compounds were plated in a 10-point dose-response and re-evaluated in the HTS assay. True positives were defined as any compound yielding a well-behaved, saturating sigmoidal curve in the dose-response assay. The ROC curve, shown in Fig 1D, demonstrated that the assay has good discriminatory power, with an area under the curve (AUC) of 0.89 (a perfect assay would have an AUC 1.0, whereas a random assay has an AUC of 0.5). Based on this analysis, a cut-off value of > 65% inhibition was chosen, resulting in 2,703 primary hits with an expected true positive rate of 85%. It is worth noting that a significant number of true hits likely remain in the group of compounds exhibiting growth inhibition of lower the 65% cut-off activity, and these compounds were not considered in this manuscript.

### Secondary confirmation of antileishmanial activity

**Fig 2:**
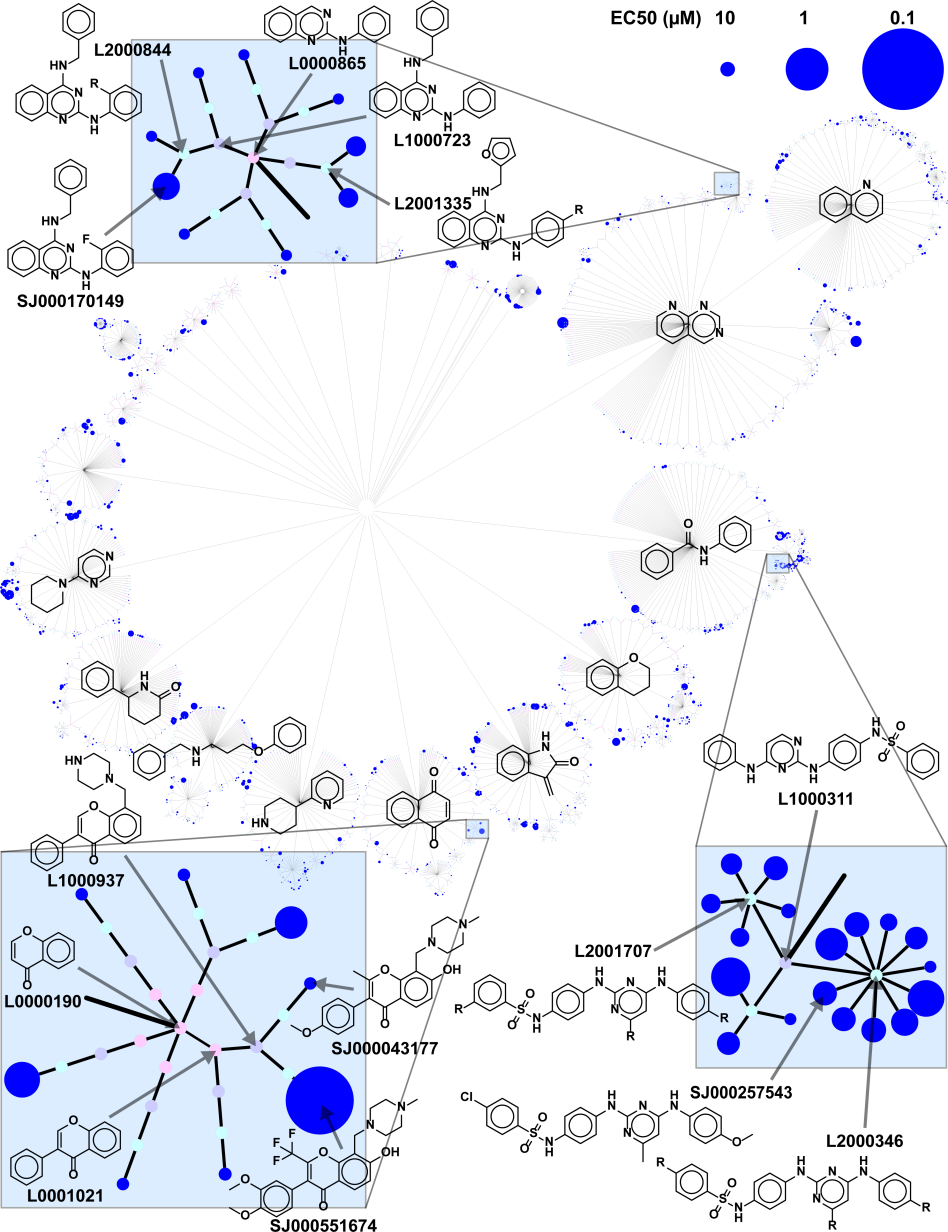
Identification and structure clustering of antileishmanials identified from the HTS campaign. Chemical structure network graph showing the structural clusters and antileishmanial potencies of the 2,703 primary screen hits. Topologically similar molecules cluster together in the branches of the network. The graph was constructed by first abstracting molecules to scaffolds and then to cores using the Murcko algorithm [35]. Each of these structural entities is represented as a node, and nodes are connected via edges according to topological relationships, with closeness being defined using the Tanimoto coefficient [57]. The colors of the nodes represent increasing functionalization of the scaffold core structures, starting from pink (most basic core scaffold) to blue (fully functionalized hit structure). The highly branched structure of the full network graph indicates that the 2,703 compounds are organized into clusters of clusters: cores are well sampled by multiple scaffolds, and the cores themselves are grouped into families of related chemotypes. Three potent core scaffolds are blown up to provide greater detail and highlight the structure activity relationships that existed within the screening collection: Top blue box: 2,4-diaminoquinazoline; bottom right blue box: 2,4-diaminopyrimidine; bottom left blue box: 4*H*-chromen-4-one.

To confirm the activity of the primary hits and improve confidence that they would be reasonable starting points for drug development, a variety of secondary screens and analyses were employed (Fig 1A). First, EC_50_ values were determined against the promastigotes using a 10-point dose-response, run in triplicate, with concentrations ranging from 0.0005–25 μM. Compounds that reproducibly exhibited EC_50_ activity lower than 2 μM were considered validated hits. In parallel, mammalian cell growth inhibition was determined using *in vitro* proliferation assays with normal human fibroblasts (BJ cells). Compounds inhibiting proliferation of BJ cells at concentrations lower than 20 μM were deprioritized. To further triage the hits, we carried out a chemical structure analysis for the 2,703 primary hit compounds utilizing topology mapping and clustering methodology [16]. We identified a wide range of chemotypes, including several scaffolds with potential structure-activity relationships (SARs), based on their dose response activity (Fig 2). Validated hits were then culled by eliminating scaffolds with less favorable drug development properties such as charged planar structures, reactive electrophilic warheads, known pan-assay interference motifs (PAINS) [47], and compounds displaying gross rule of five noncompliance [48]. Finally, we prioritized scaffolds with the possibility of facile chemical modification to generate a substantial number of structural analogs for future SAR and structure-property relationship (SPR) studies.

Among the 2703 hits, 230 compounds exhibited both an EC_50_ of < 2 μM for *L*. *mexicana* promastigotes and a TI > 5 (based on mammalian fibroblast toxicity). These were chosen as candidates for further study. From these 230 compounds, we were able to repurchase 113 from commercial vendors. These compounds were characterized for purity by ultra-performance liquid chromatography using ultraviolet spectroscopy and evaporative light scattering detection [49] and identity by mass spectrometry. All validated compounds were profiled for activity against intracellular amastigotes, the disease-causing stage of the life cycle. Intracellular amastigote activity was determined using a strain of *L*. *mexicana* in which the *Renilla* luciferase gene was integrated into the rRNA locus [43], allowing robust expression for measuring amastigote growth within cultured macrophages [50]. All 113 compounds were applied at 1 μM concentration for 96 h to J774A.1 macrophages infected with *L*. *mexicana* luciferase-expressing parasites. Of the compounds tested 55 inhibited amastigote growth by > 70%.

**Fig 3:**
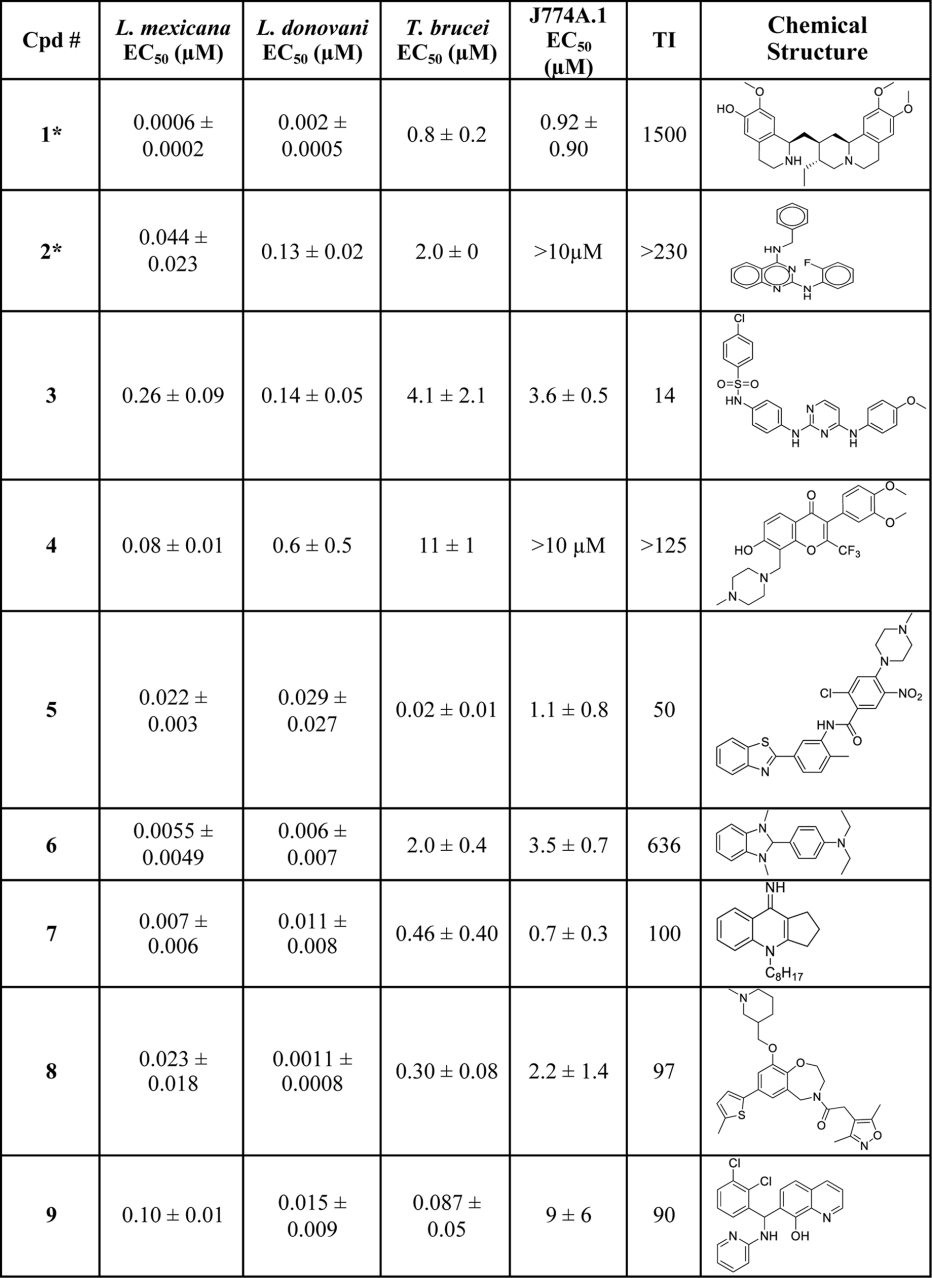
Top hits for inhibition of growth of *L*. *mexicana* amastigotes. EC_50_ values represent the mean ± standard deviation for n = 2 and were calculated from dose-response curves against intracellular amastigotes of *L*. *mexicana* and *L*. *donovani*, the bloodstream form of *T*. *brucei*, and the host macrophage J774A.1. TI was calculated as EC_50_ J774A.1/EC_50_ *L*. *mexicana* amastigotes. None of the nine compounds inhibited proliferation of normal fibroblasts (BJ cells) at 20 μM. *Exact compound has been previously reported as exhibiting antileishmanial activity. For J774.A5 macrophages, compounds were tested up to 10 μM concentration, and those that showed no inhibition of growth were reported to have an EC_50_ value of >10μM.

Next, we generated dose-response curves for these 55 compounds against intracellular amastigotes and independently against J774A.1 macrophages to establish the relative potency of each compound against the pathogen and its host cell. Those that had EC_50_ values < 1 μM and TI values > 10 for macrophages were selected from the 55 compounds, as suggested for lead identification for leishmaniasis [12], and several compounds were then removed due to known biological liabilities of scaffolds (manual curation, Fig 1). The nine remaining compounds, each representing a unique chemical scaffold (compounds **1**–**9**, Fig 3), were designated top hits (Fig 1).

Notably, this screening strategy successfully identified several known antileishmanial scaffolds, including compounds **1**, **2**, **3,** and **4**, thus providing further validation of the screen. The alkaloid cephaeline (**1**), a known irritant of gastric mucosa and component of ipecac, has been shown to be potent against *L*. *mexicana* and *L*. *donovani* intracellular amastigotes [51]. Another known scaffold, the quinazoline-2,4-diaminoquinazolines, represented by compound **2**, has been studied extensively and shown to have activity against *L*. *donovani*, and *L*. *amazonensis* [52, 53]. Compound **2** is also present in the malaria box of compounds active against *Plasmodium falciparum* and has been shown to have activity against *L*. *infantum* [54]. We also found a member of the 2,4-diaminopyrimidine scaffold, compound **3**, some of which are selective against *L*. *major* amastigotes, with EC_50_ values in the low μM range and in once case with a therapeutic index (TI) as high as 130 [55]. The compounds in that study share the 2,4-diaminopyrimidine scaffold with compound **3**, but they differ in having a benzyl substitution at the 5 position of the pyrimidine ring rather than modifications on the 2- and 4-amino substituents that are present in compound **3**. Finally, various 4*H*-chromen-4-ones, similar to compound **4**, are active against *L*. *major* [56]. Of the validated scaffolds included in the HTS campaign, the three that exhibited the widest SAR range (7–88 fold) were the 2,4-diaminoquinazolines, 2,4-diaminopyrimidines, and 4*H*-chromen-4-ones (Fig 2).

Potency of the nine compounds against intracellular amastigotes of *L*. *donovani* was also quantified to assess each compound’s potential to control this agent of fatal visceral leishmaniasis (Fig 3). We have recently reported that compound **5** is also potent against another kinetoplastid parasite, the bloodstream form of *Trypanosoma brucei* (EC_50_ value of 0.027 μM) [58] and active *in vivo* in a murine model of African trypanosomiasis (manuscript in preparation). Thus, we also evaluated the other compounds for activity against the related pathogen *Trypanosoma brucei*. As noted for the broad spectrum kinetoplastid proteasome inhibitor GNF6702 [32], compounds exhibiting activity against multiple parasites are especially interesting, as such scaffolds can be explored for therapies against multiple neglected parasitic diseases.

All nine compounds were potent (EC_50_ < 0.6 μM) against both the *L*. *mexicana* and *L*. *donovani* intracellular amastigotes. Often, potency correlated well between the two species, although there were significant differences for some compounds (e.g., compounds **2**, **4**, **8**, and **9**). While none of the compounds affected the proliferation of BJ cells at concentrations as high as 20 μM, most of the compounds reduced viability of macrophages with half-maximal lethal dose (LD_50_) values around 1–10 μM. Only compounds **2** and **4** demonstrated no reduction in viability in dose-response studies against the host macrophage J774A.1, suggesting these compounds may afford the best selectivity for inhibiting parasite growth relative to toxicity toward the host macrophage or other mammalian cells. Thus, all of the nine compounds tested afforded favorable therapeutic indices (> 50), except compound **3**. Notably, compounds **5**, **8**, and **9** exhibited good potency (< 0.3 μM) against bloodstream form *T*. *brucei*. To determine whether any of the top hits might also be effective against the related kinetoplastid parasite *T*. *cruzi*, we performed dose-response curves with compounds **4**, **5**, **8**, and **9** against epimastigotes and found either no inhibition (**4**, n = 3) or EC_50_ values of 0.086 ± 0.03 μM (**5**, n = 4), 0.33 μM (**8**, n = 1), and 2.1 ± 0.07 μM (**9**, n = 2), respectively. Hence, each of these latter scaffolds is of potentially high interest for development of drugs against multiple species of kinetoplastid parasites.

Together, these data suggest the seven compounds not previously reported to possess antileishmanial activity (only **1** and **2** have been documented previously) can be good starting points for discovering new antileishmanials. Herein, we chose to further profile compounds **4** and **5**, representing the 4*H*-chromen-4-ones and *p*-chloronitrobenzamides scaffolds, respectively. Compound **4** was chosen for its distinct lack of toxicity against host macrophages and compound **5** was chosen for its cross-species potency. We suggest that similar studies could be undertaken using the other validated compounds from our two-stage phenotypic screening campaign.

### *In vitro* Absorption, Distribution, Metabolism, and Excretion (ADME)

In order to evaluate compounds **4** and **5** for *in vivo* studies, we measured the *in vitro* ADME physiochemical properties likely to be predictive of oral bioavailability (Table 1). First, we looked at solubility in an aqueous buffer (pH = 7.4) and ability to cross an artificial (parallel artificial membrane permeability, PAMPA) or cellular (Caco-2) membrane. Compound **4** exhibited good solubility (67 μM) and moderate membrane permeability (Table 1) suggesting a high predicted absorption across the intestinal epithelium (~85%), and low probability of being a substrate of the drug resistance pumps expressed by Caco-2 cells (efflux ratio < 2). Compound **5** showed moderate permeability in both the PAMPA and Caco-2 assays as well as an acceptable efflux ratio of 1.92 (Table 1). Compound **5** exhibits low aqueous solubility (0.3 μM) but we anticipated that this could be compensated by formulation for delivery [59]. Next, we investigated the stability of both compounds in simulated gastric fluid and in microsomal models of oxidative metabolism. Both compounds exhibited high stability in simulated gastric fluid (t_1/2_ > 24 h) and demonstrated good metabolic stability (t_1/2_ > 4 h for all species) in liver microsome preparations from mouse, rat, and human. Compounds **4** and **5** also showed modest (<50%) binding to mouse plasma proteins, below the level of the positive control drug propranolol (Table 2). Criteria that have been suggested as promising for an orally bioavailable compound include: aqueous solubility > 1 μM but ideally > 100 μM [60], PAMPA permeability coefficient of > 1x10^-5^ cm/sec represents high permeability, Caco-2 cell permeability coefficient of > 1x10^-6^ cm/sec [60], Caco-2 cell efflux ratio < 2 represents no efflux, gastric stability > 24 h, t_1/2_ in microsomes > 30 min [12]. However, these values only represent broad guidelines, and many efficacious drugs violate them. Overall, the *in vitro* ADME data suggest that the scaffolds of compounds **4** and **5** would be appropriate for development into orally bioavailable antileishmanial compounds.

**Table 1:** *In vitro* ADME for compounds 4 and 5 and drug controls.

Compound	Solubility^*a*^ (μM)	PAMPA^*a*^ (10^-5^cm/s)	Caco-2^*a*^ (A→B) (10^-5^cm/s)	Caco-2^*a*^ (B→A) (10^-5^cm/s)	Caco-2^*a*^ (ratio)	Gastric Stability t_1/2_ (h)	Microsomal stability t½ (h)
**4**	67	37.5	4.90 ± 0.03	3.90 ± 0.07	0.80	> 48	Mouse >4 Rat >4 Human > 4
**5**	0.3	23.7	1.68 ± 0.24	3.22 ± 0.29	1.92	> 24	Mouse >4 Rat >4 Human > 4
**carbamazepine**	82.2	9.56	7.18 ± 0.12	5.65 ± 0.32	0.79	> 24	Mouse >4 Rat >4 Human > 4
**digoxin**	80.3	ND	0.461 ± 0.046	2.71 ± 0.21	5.87	> 24	Mouse >4 Rat >4 Human > 4
**albendazole**	3.8	20.4	ND	ND	ND	> 24	Mouse 0.7±0.04 Rat 1.62±0.11 Human > 4
**verapamil**	70.0	150	ND	ND	ND	> 24	Mouse 0.8±0.1 Rat 0.4±0.8 Human 0.6±0.1

^*a*^Experiments performed at pH 7.4. Microsomal stability was tested at 20 μM. Data for Caco-2 experiments represent the mean ± the standard deviations for three separate runs. The numbers for PAMPA and Caco-2 assays represent permeability coefficients. Results reported as means and standard deviations represent 3 replicate experiments. ND indicates not determined.

**Table 2:** Plasma protein binding for compounds 4, 5, and the control drug propranolol.

Compound	Conc. (μM)	Mouse plasma protein binding (%)
Propranolol HCl	4	71.6 ± 9.3
**4**	20	46.6 ± 8.7
	4	40.1 ± 6.4
	0.8	46.0 ± 11.1
**5**	20	41.4 ± 7.6
	4	40.9 ± 4.5
	0.8	35.0 ± 8.8

### *In vivo* pharmacokinetics

**Fig 4:**
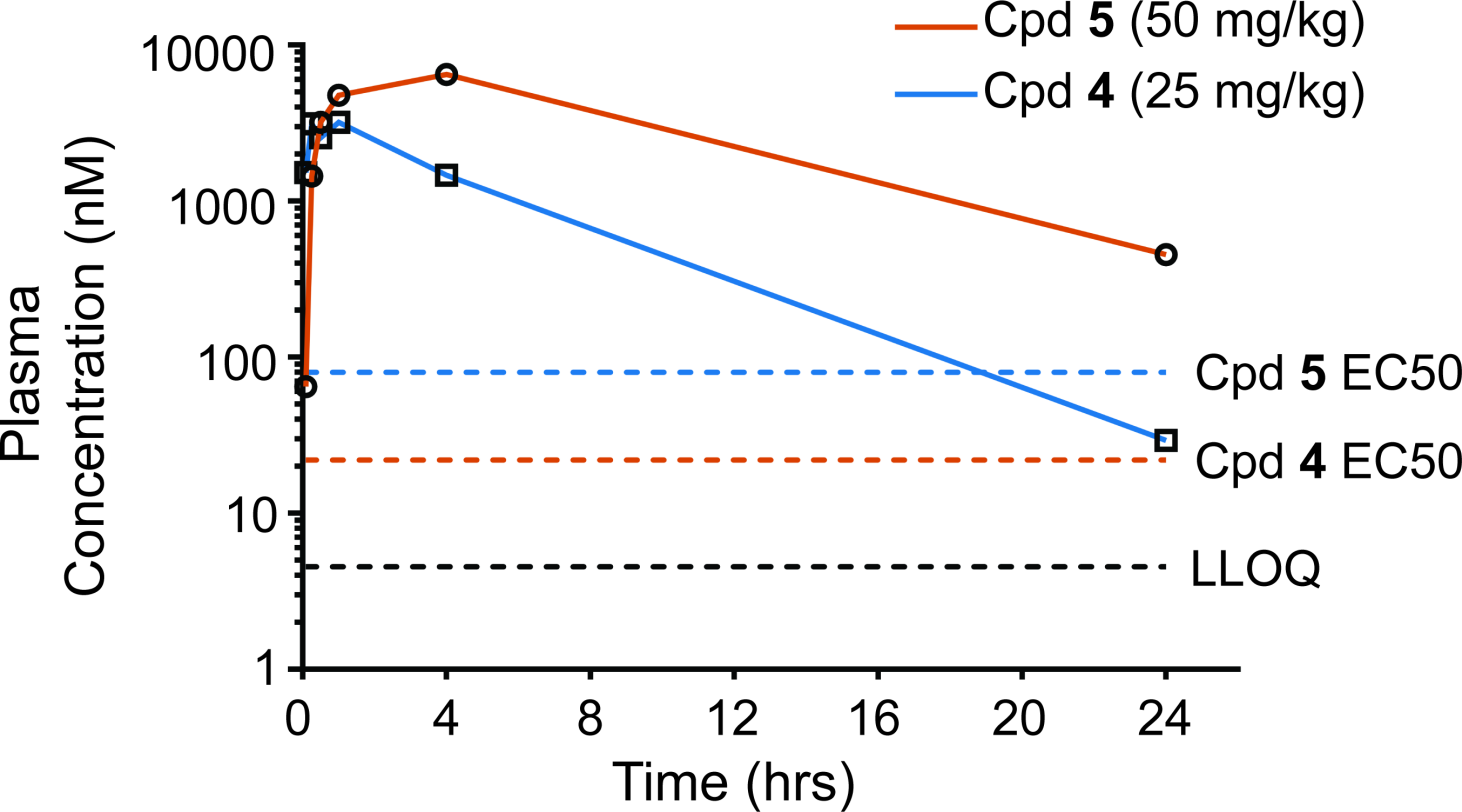
*In vivo* pharmacokinetic profiling of compounds 4 and 5. Murine pharmacokinetic studies for compound **4** and **5** delivered *per os* (25 mg/kg and 50 mg/kg respectively).

To further evaluate the potential of **4** and **5** *in vivo*, we performed preliminary single oral dose pharmacokinetic studies in mice. Following a single oral gavage (PO) of **4** in mice at 25 mg/kg (Fig 4) the plasma concentration remained above its EC_50_ of 0.08 μM for approximately 20 h. Compound **4** reached a peak plasma concentration (*C*_max_) of 3.2 μM within 1 h (*t*_max_) of dosing, afforded an AUC of 16.7 μM.h, and an elimination half-life (*t*_1/2_) of 3 h (Table 3). Following PO dosing of **5** at 50 mg/kg (Fig 4, Table 3), the plasma concentration remained above the EC_50_ of 0.022 μM for roughly 48 h, with a *C*_max_ of 6.49 μM, a *t*_max_ of 4 h, an AUC of 83.2 μM*h, and a t_1/2_ of 7.1 h. Thus, both compounds exhibited good plasma exposure and sustained plasma concentrations above an efficacious dose (EC_50_) for more than 12 h following a single oral gavage dosing using our standard formulation (10/10/40/39, EtOH/PG/PEG/PBS (7.4) (v/v) and 1% (w/v) HβCD). These results strongly suggested that both compounds were appropriate candidates for efficacy evaluation in the murine model of cutaneous leishmaniosis.

**Table 3:** Pharmacokinetic parameters for compound 4 and 5 based on oral administration in mice.

Compound	Dose (mg/kg)	t_½_ (h)	*C*_max_ (μM)	*t*_max_ (h)	AUC (μM*h)	CL (L/h/kg)	V_d_ (L/kg)
**4**	25	3.2	3.2	1	16.7	3.13	14.2
**5**	50	7.1	6.5	4	83.2	1.15	11.8

t_1/2_ is the compound half-life in plasma, *C*_max_ is the maximum concentration, *t*_max_ is the time the compound takes to achieve the maximum plasma concentration, AUC is area under the curve, CL is clearance rate, V_d_ is volume of distribution.

### Determination of maximum tolerable doses

Next, we sought to determine the allowable dosing range for our efficacy model by carrying out dose-ranging tolerability studies. When compound **4** was dosed by oral gavage at 50 mg/kg, half of the animals exhibited seizure-like behavior. Blood chemistries revealed a very low glucose level in plasma (23–40 mg/dl for treated mice compared to 185–251 mg/dl for untreated mice). This observation might suggest blockage of a kidney and/or an intestinal glucose transporter. When we repeated the same experiments at 25 mg/kg, no seizures were seen and the glucose level of each animal remained within normal limits at both *C*_max_ and *C*_min_. Daily oral administrations of **4** at 25 mg/kg were well-tolerated in all study animals, no significant changes in either clinical chemistry or complete blood counts were observed, and there were no other test article-related effects noted in the liver or any other tissues. For compound **5** dosed at 50 mg/kg in mice, a 10-day toxicity study revealed that animals reduced food intake and lost more than 10% of their weight overtime. The observed suppressed appetite was resolved by dose reduction to 30 mg/kg. Thus, we employed 25 mg/kg of **4** and 30 mg/kg of **5** in the efficacy model. No weight loss or other toxicity was observed at these doses.

### *In vivo* efficacy studies

**Fig 5:**
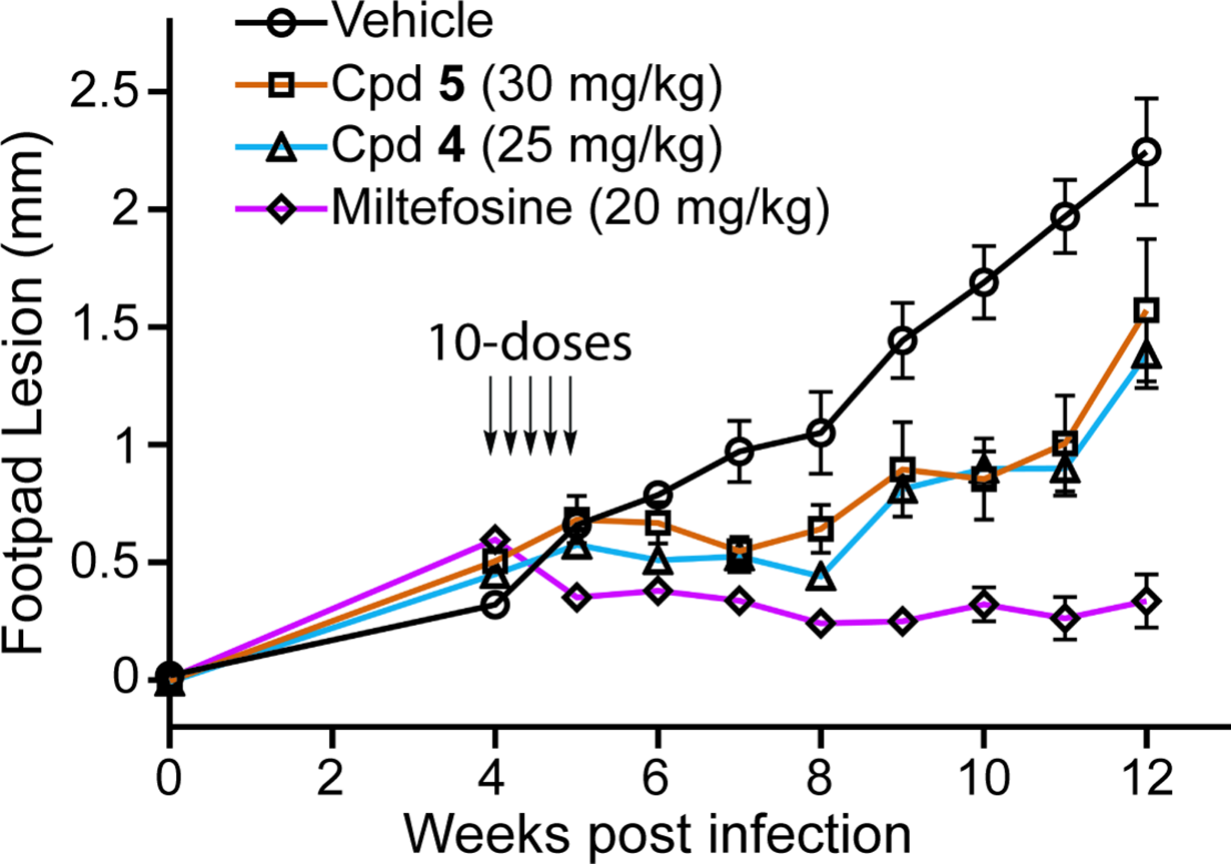
*In vivo* efficacy for controlling cutaneous lesion progression in the mouse. Mice (5 per cohort) were infected with *L*. *mexicana* promastigotes on day 0; by week 4 after infection, cutaneous lesions had grown to ~0.5 mm width. Compound **4** (triangles), compound **5** (squares), or miltefosine (diamonds) were delivered daily by oral gavage for 10 sequential days. One cohort of mice (circles) received vehicle alone. Measurements are plotted as the mean ± standard deviation.

Next we assessed the potential of compounds **4** and **5** to control disease in a murine model of cutaneous leishmaniasis [30]. We infected BALB/c mice with *L*. *mexicana* via footpad injections on day zero, allowed incipient lesions to develop for four weeks, and then treated cohorts of five animals with each compound for 10 consecutive days by oral gavage. In addition, five mice were treated with 20 mg/kg of the only orally available approved antileishmanial drug, miltefosine, as a positive control and with vehicle alone as a negative control. Footpad widths were measured from 4–12 weeks post-infection (Fig 5). For vehicle-treated mice, the lesions grew steadily up to 2 mm width, at which time mice were euthanized. Miltefosine reduced lesion size from the initial dimension and was able to maintain growth inhibition for eight weeks following treatment. Both compounds **4** and **5** controlled lesion size at dimensions similar to that at the time of compound dosing (4 weeks) until week 9, well after stopping oral administration. After week 9, the footpad lesions began to increase in size. Hence, oral dosing of both compound **4** and **5** controlled disease progression during the dosing period and for a significant period of time after dosing stopped but neither was as efficacious as miltefosine. The partial control of virulence exhibited by our HTS hits, without any optimization, strongly suggests both compounds are novel early leads for the development of orally available antileishmanials. Given the synthetic tractability of these scaffolds [58, 61], we envision a rapid timeline for the development of optimized leads with enhanced therapeutic properties.

## Discussion

High throughput phenotypic screening offers a powerful tool to discover therapeutically relevant leads for drug discovery [62]. The HTS campaign described in this paper represented part of a larger effort to identify selective inhibitors of hexose transporters from various parasitic protozoa [45], including a transgenic strain of *L*. *mexicana*, Δ*lmxgt1-3*[p*LmxGT2*] [63]. The results reported here began as a second, adventitious outcome of that screen, where we identified 2,703 compounds that significantly inhibited the growth of promastigotes of the Δ*lmxgt1-3* parasites employed as the cellular expression vehicle for the hexose transporters. In this study, we leveraged this secondary outcome to identify novel orally available antileishmanials. We emphasize that since most of the top hits against *L*. *mexicana* are also potent against an agent of lethal visceral leishmaniasis, *L*. *donovani* (Fig 3), this screen is of potential therapeutic value for both cutaneous and visceral leishmaniasis.

The initial screen against the *L*. *mexicana* promastigote form of the parasite was highly robust with a median *Z* value of 0.81 and an AUC of 0.893 for the ROC curve. In addition to the antileishmanial compounds that were carried through the secondary validation assays (*vide supra*), the HTS identified a variety of inhibitors known to be active against various *Leishmania* strains: crystal violet (EC_50_: 0.29 μM) [64], disulfiram (EC_50_: 0.50 μM) [65], thiram (EC_50_: 1.77 μM) [65], actinomycin D (EC_50_: 0.36 μM) [28], anisomycin (EC_50_: 0.58 μM) [66], and avicin (EC_50_: 1.50 μM) [16]. The rediscovery of these known inhibitors provided another level of validation and confirmed the screen’s ability to identify active antileishmanial compounds.

Our motive behind the sequential screening of promastigotes followed by amastigotes was to eliminate promastigote-specific hits. In the process we identified multiple hits that were potent inhibitors of both promastigote and amastigote growth and removed compounds that inhibited growth of either host macrophages (J774A.1) or normal fibroblasts (BJ cells) (Fig 3). Hence, while there has been much discussion about the relative merits of screens employing promastigotes, axenic amastigotes, and intracellular amastigotes (see Introduction), the sequential approach employed here sidesteps that debate and identified multiple scaffolds with potential for further development toward orally bioavailable antileishmanial drugs.

Three scaffolds from the sequential screen stood out as promising candidates due to the wide range of SAR inherent in our screening data set (Fig 2), the high potency of certain exemplars, and good TI values (Fig 3): 2,4-diaminoquinazolines (**2**), 2,4-diaminopyrimidines (**3**), and 4H-chromen-4-ones (**4**). The 2,4-diaminoquinazolines have been disclosed previously as potential antileishmanials [52, 53, 67]. This scaffold has been explored by medicinal chemistry, and one compound was identified that exhibited an EC_50_ of 0.15 μM against *L*. *donovani* amastigotes and a TI of 100 [52, 53, 67]. These studies also demonstrated that *Leishmania* dihydrofolate reductase (DHFR) is inhibited by 2,4-diaminoquinazolines, highlighting this essential enzyme as one target for this class of antileishmanials.

Similarly, 2,4-diaminopyrimidines have been shown to have μM potency against *Leishmania* amastigotes [55]. Compound **3** is more potent than the previously studied 2,4-diaminopyrimidines [55], and it has substitutions on the 2,4-amino groups, unlike previously characterized 2,4-diaminopyrimidines. These results suggest that substitution at these positions may be important for potency and imply that additional modifications at these sites may be worth exploring. Furthermore, 2,4-diaminopyrimidines are structurally related to classical DHFR inhibitors such as pyrimethamine and trimethoprim [55] that also selectively inhibit the essential *Leishmania* DHFR, providing a potential molecular target for this family of antileishmanials. However, two distinct enzymes in *L*. *major*, DHFR and pteridine reductase 1 (PTR1), can reduce folate, and amplification of the *PTR1* gene can confer methotrexate resistance upon the parasite by metabolically circumventing inhibition of DHFR by this antifolate [68]. Hence, effective inhibitors of DHFR may also need to inhibit PTR1, thus complicating chemotherapy against this target.

4*H*-chromen-4-ones [56], and related chroman-4-ones [69], have been demonstrated to have activity against both *T*. *brucei* and *L*. *major*, and they bind to and inhibit the critical [70] enzyme PTR1 that is present in kinetoplastid parasites, but not in mammals. These results with structurally related compounds suggest that PTR1 may be a principle target of compound **4**. However, the compounds tested in this previous work had an aromatic substituent at the 2 position of the chromen-4-one ring rather than at the 3 position. Those compounds exhibited much lower potency against *T*. *brucei* and *L*. *major*, with EC_50_ values in the micromolar range, compared to compound **4** against *L*. *mexicana* or *L*. *donovani* amastigotes (Fig 3). Furthermore, compound **4** has lower toxicity, a higher TI (Fig 3, no inhibition of J774A.1 macrophages up to 10 μM concentration), and greatly superior pharmacokinetic properties (Fig 4, Table 3) compared to compounds tested by Borsari et al. [56], where the top hit exhibited a half-life of 7.6 min in mice. These observations suggest that structural features present in compound **4** may provide a route for developing this scaffold toward more optimal lead compounds against *Leishmania* parasites. Variants of the isoflavone scaffold present in **4** have been employed as dietary supplements and are known to have phytoestrogen and antioxidant properties [71]. Thus, there has been a long-standing interest in the development of synthetic routes to access these desirable properties [72, 73]. More recently, rapid synthetic routes to access highly substituted hydroxylated isoflavones such as **4** have been published [61].

Compound **5** has been identified previously [58] by our laboratory as active against all *Trypanosoma* species *in vitro* and efficacious against *T*. *congolense* and *T*. *b*. *rhodesiense in vivo* (manuscript in preparation). This scaffold is especially interesting, since it may act on a common cellular target found among kinetoplastids and could be developed as both antileishmanial and antitrypanosomal drugs. In addition, the in-house experience with the scaffold in *in vivo* models inspired confidence regarding its oral activity. Therefore, we chose to progress compounds **4** and **5** for further *in vitro* ADME and *in vivo* pharmacokinetic and pharmacodynamic testing. We note that compounds **8** and **9** also exhibit significant potency toward *T*. *brucei* and may therefore be of special interest for future investigations.

Compounds **4** and **5** were both able to partially control the size of an incipient cutaneous lesion when delivered orally at 25–30 mg/kg for 10 days, compared to mice that received vehicle alone. However, they were not as efficacious as the currently employed oral drug miltefosine. This efficacy *in vivo* indicates that improvements will be required to further address the potential of these scaffolds for drug development. In particular, analogs that exhibit higher potency and/or lower toxicity in animals may achieve greater efficacy or allow higher dosing. The ability to extensively modify both scaffolds by medicinal chemistry offers the potential to generate libraries of analogs of each lead whose members can then be tested for improved ADME, PK, toxicity, and *in vivo* efficacy in animal models of both cutaneous and visceral leishmaniasis. Additionally, the combination of potency, selectivity, and identification of DHFR as a potential molecular target all suggest that further exploration of the 2,4-diaminoquinazoline and 2,4-diaminopyrimidine scaffolds, represented by compounds **2** and **3** respectively, may be warranted. Overall, this work demonstrates how sequential screening of promastigotes, which are especially amenable to HTS assay development, followed by hit validation in the disease causing intramacrophage amastigotes can be used to successfully identify novel antileishmanial scaffolds. The promising pharmacokinetic profile and significant *in vivo* efficacy of our newly identified scaffolds strongly suggests that additional medicinal chemistry optimization may yield orally available anti-parasitic drugs.

## Supporting information

S1 Appendix Additional information regarding materials and methods employed. (DOCX)
