# Supplementary material for: Discovery of novel, orally bioavailable, antileishmanial compounds using phenotypic screening
Source: PLoS Negl Trop Dis. 2017 Dec 29;11(12):e0006157. doi: 10.1371/journal.pntd.0006157 (PMC5764437; doi:10.1371/journal.pntd.0006157)
Supplement: S1 Appendix — (DOCX) [file pntd.0006157.s001.docx]

**S1 Appendix.** Additional information regarding materials and methods employed.

**Phenotypic Screening Identifies Novel Orally Bioavailable Antileishmanial Compounds**

**Short title:** Novel Antileishmanial Compounds

Diana Ortiz^1^*, W. Armand Guiguemde^2^*, Jared T. Hammill^2,3^, Angela K. Carrillo^2^, Yizhe Chen^2,3^, Michele Connelly^2^, Kayla Stalheim, Carolyn Elya, Alex Johnson, Jaeki Min^2^, Anang Shelat^2^, David C. Smithson^2^, Lei Yang^2^, Fangyi Zhu^2^, R. Kiplin Guy^2,3‡^, Scott M. Landfear^1‡^

^1^Department of Molecular Microbiology & Immunology, Oregon Health & Science University, Portland, Oregon, United States of America

^2^Department of Chemical Biology and Theraputics, St. Jude Children’s Research Hospital, Memphis, Tennessee, 38105 USA

^3^Present address: Department of Pharmaceutical Sciences, University of Kentucky, Lexington, KY 40508

*These authors are designated as joint first authors

^‡^These authors are designated as joint corresponding authors

**Liver microsomal stability**

For microsomal stability assays [1] 0.633 mL of mouse liver microsome (20 mg/mL, female CD9 mice, Fisher Scientific, #NC9567486) or human liver microsome (200 pooled mix gender, Fisher Scientific #50-722-552) was mixed with 0.051 ml of 0.5M EDTA solution and 19.316 ml potassium phosphate buffer (0.1M, pH 7.4, 37°C) to make 20 ml of liver microsome solution. One part of 10 mM DMSO compound stock was mixed with 4 parts of acetonitrile to make 2 mM diluted compound stock in DMSO and acetonitrile. 29.1 μL diluted compound stock was added to 2.3 mL liver microsomal solution and vortexed to make microsomal solution with compound. 180 μL of the microsomal solutions with different compounds were dispensed into respective rows of a 96-well storage plate (pION Inc., #110323). For the 0 h time point, 450 μL pre-cooled (4 ºC) internal standard (10 μM warfarin in methanol) was added to the first three columns before the reaction was initiated. 1.25 ml of microsome assay solution A (Fisher Scientific, #NC9255727) was combined with 0.25 ml of solution B (Fisher Scientific, #NC9016235) in 3.5 ml of potassium phosphate buffer (0.1 M, pH 7.4). 45 μL of this A+B solution was added to each well of the 96-well storage plate (reaction plate). Liquid in the first 3 columns was moved to another storage plate (quenched plate). The reaction plate was then sealed, incubated at 37 ºC, and shaken at a speed of 60 rpm. Time points (0.5 h, 1 h, 2 h) were taken. At each time point, 450 μL pre-cooled internal standard was added to 3 rows in the reaction plate, and the liquid was then transferred to the quenched plate. The quenched plate was then centrifuged (model 5810R, Eppendorf, Westbury, NY) at 4000 rpm for 20 min. 200 μL supernatant was transferred to a 96-well plate and analyzed by ultra-performance liquid chromatography-mass spectrometry (UPLC-MS) (Waters Inc.). The compounds and internal standard were detected by selected ion recording (SIR). The log peak area ratio (compound peak area / internal standard peak area) was plotted against time and the slope was determined to calculate the elimination rate constant [k = (-2.303) x slope]. The half-life was calculated as t_1/2_ = 0.693 / k.

**Solubility**

Solubility assays were carried out on a Biomek FX lab automation workstation (Beckman Coulter, Inc., Fullerton, CA) using μSOL Evolution software (pION Inc.). Compound stock (10 mM in DMSO, 10 μL) was added to 1-propanol (190 μL) to make a reference stock plate. Reference stock solution (5 μL) was mixed with 1-propanol (70 μL) and citrate phosphate buffered saline (pH 7, 75 μL) to make the reference plate which was then measured with UV detection. Test compound stock (10 mM, 6 μL) was added to citrate phosphate buffered saline (594 μL) in a 96-well storage plate and mixed. The storage plate was sealed and incubated at room temperature for 18 h. The suspension was then filtered through a 96-well filter plate (pION Inc.). Filtrate (75 μL) was mixed with 1-propanol (75 μL) to make the sample plate, and the UV spectrum (250 nm −500 nm) of the sample plate was recorded. Calculation of solubility was carried out using μSOL Evolution software based on the AUC of the UV spectrum of the sample plate and the reference plate. All compounds were tested in triplicate.

**Permeability assay**

Parallel Artificial Membrane Permeability Assay (PAMPA) was conducted by Biomek FX lab automation workstation (Beckman Coulter, Inc.) and PAMPA evolution 96 command software (pION Inc.). A 3 μL aliquot of 10 μM test compound stock in DMSO was mixed with 597 μL of isotonic citrate phosphate buffered saline to make diluted test compound. Diluted test compound (150 μL) was transferred to a UV plate (pION Inc.) and the UV spectrum was read as the reference plate. The membrane on a pre-loaded PAMPA sandwich (pION Inc.) was painted with 4 μL GIT lipid (pION Inc.). The acceptor chamber was then filled with 200 μL acceptor solution buffer, (pION Inc.), and the donor chamber was filled with 180 μL diluted test compound. The PAMPA sandwich was assembled, placed on the Gut-Box and stirred for 30 min. The Aqueous Boundary Layer was set to 40 μm for stirring. The UV spectrum (250-500 nm) of the donor and the acceptor solutions were recorded. The permeability coefficient was calculated using PAMPA Evolution 96 Command software (pION Inc.) based on the AUC of the reference plate, the donor plate and the acceptor plate. All compounds were tested in triplicate.

**Caco-2 permeability assay**

High throughput Caco-2 permeability measurements were performed on the Transwell 0.4 µm polycarbonate membrane 96-well system with modified methods [2]. Caco-2 cells were maintained at 37 °C in a humidified incubator with an atmosphere of 5% CO_2_. The cells were cultured in 75 cm^2^ flasks with Dulbecco’s Modified Eagle’s Medium containing 10% fetal bovine serum, 1% non-essential amino acids, 100 units/ml of penicillin, and 100 μg/mL of streptomycin. The Caco-2 cells were seeded onto inserts at a density of 2×10^4^ cells/insert. The medium in the wells was exchanged every other day, and the trans epithelial electrical resistance (TEER) value was measured using an epithelial volt-ohm meter (Millipore). Caco-2 cells were grown for 7 days to reach consistent TEER values (typically 2000 ohms greater than initial value when cells are first seeded into transwells), indicating that the cells had formed a confluent polarized monolayer.

For transport experiments, each cultured monolayer on the 96-well plate was washed twice with a transport buffer (Hank’s Balanced Salt Solution plus 25 mM HEPES, pH 7.4). The permeability assay was initiated by the addition of each compound solution (10 μmol/L) into inserts (apical side, A) or receivers (basolateral side, B). The Caco-2 cell monolayers were incubated for 2 h at 37 °C. Fractions were collected from receivers (if apical to basal permeability) or inserts (if basal to apical permeability), and concentrations were assessed by UPLC/MS (Waters). All compounds were tested in triplicate.

The A→B and B→A apparent permeability coefficients (P_app_) of each compound were calculated using the equation, P_app_=dQ/dt×1/AC_0,_ where dQ/dt equals the flux of a drug across the monolayer, A equals the total insert well surface area, and C_0_ is the initial concentration of substrate in the donor compartment. The efflux ratio was determined by dividing the P_app_ in the B→A direction by the P_app_ in the A→B direction. An efflux ratio >2 suggested that a given substrate was actively transported across the membrane.

**Stability in simulated gastric fluid**

Compound stocks were 10 mM in DMSO. The internal standard was 5 µM warfarin in methanol. 1.4 ml concentrate HCl (37%), 0.4 g NaCl and 0.64 g pepsin were added to 198 ml deionized water to make simulated gastric fluid (SGF) (pH 1). 1 ml SGF was added to a 1.1 ml 96-well storage plate and mixed well with 10 µl compound stock. The plate was then incubated at 37 ºC and shaken at 60 rpm. Samples were taken at 0 min, 15 min, 30 min, 1 h, 2 h, 4 h, 8 h and 24 h. At each time point, 100 µl was removed to a 1 ml storage plate and 300 µl internal standard was added to quench the reaction. The plate was then centrifuged at 4000 rpm for 15 min and supernatant was analyzed by UPLC-MS. The compound was detected by SIR and quantification was based on peak area of test compound and the internal standard.

**Mouse plasma protein binding assay**

A Rapid Equilibrium Dialysis (RED) Plate (Thermo Scientific, catalog #, PI-90007) was used to determine the percentage of compound binding to mouse plasma proteins (Thermo Scientific, catalog #, 50642085). The positive control for this experiment was propranolol HCl (295.807 g/mol) and the internal standard was warfarin in methanol (2 μM). 10 mM stocks of compound in DMSO were diluted with DMSO and acetonitrile to three different intermediate concentrations: high (2 mM), medium (0.4 mM) and low (0.08 mM) concentration in DMSO:acetonitrile (1:4, v:v). 10 mM stock of propranolol in DMSO were diluted to 0.4 mM concentration in DMSO:acetonitrile (1:4 v:v).

In 10 Eppendorf tubes, the control (10 μL) or each of three compound dilutions (10 μL) were each added to separate aliquots of 990 μL mouse plasma. Potassium phosphate buffer (500 μL, 0.1 M, pH 7.4, 37 °C) was placed in every white well of the RED plate and each plasma/compound mixture was added to each of 3 RED wells (300 μL). The RED plate holds triplicate samples of one control (final concentration 0.4 μM) and one compound (final concentrations: 20 μM, 4 μM, 0.08 μM). The RED Plate was sealed and incubated at 37 ºC shaking at 250 rpm for 4 hours. After incubation, aliquots (50 μL) from each well in the RED plate were transferred to an assay plate (pION Inc., MA, #110323). In order to create a uniform matrix in every well of the assay plate, plasma (50 μL) was added to each of the wells that already contained buffer and potassium phosphate buffer (50 μL) was added to each of the wells that already contained plasma/compound. Pre-cooled internal standard (300 μL) was added to the assay plate to quench the reaction. The quenched plate was then centrifuged (5810R, Eppendorf, Westbury, NY) at 3000 rpm for 20 minutes. 150 uL supernatant was transferred to a 96-well plate and analyzed by UPLC-MS (Waters, Inc, Milford, MA).

The compounds and internal standard were detected by selected ion recording (SIR). The percentage of free compound [1] and bound compound [2] was calculated usng the AUC ratio of compound to warfarin from the SIR spectra. For reporting data, % Free = Concentration buffer/Concentration plasma *100 %, and % Bound = 100% - % Free.

**Pilot mouse pharmacokinetic study with compound 4**

Female BALB/c mice of 17-21 grams were purchased from Charles River Laboratories (Wilmington, MA). Food and water were provided *ad libitum*. 6 mice were administered intravenously at 5 mg/kg and the other 6 received 25 mg/kg via oral gavage. All animal received 100 ± 20 µL of compound solution in formulation (10/10/40/40: EtOH/PG/PEG400/PBS (pH 7.4), v/v/v) for IV and 10/10/40/39 EtOH/PG/PEG400/PBS (pH 7.4), 1 % w/v HβCD for oral delivery. 0.1 ml blood was collected retro-orbitally from a different mouse within each dosage group at 5 min, 15 min, 30 min, 1 h, 4 h, and 24 h. Animals were euthanized via cardiac puncture at 48 h post injection. Blood samples were treated with 10 µL of EDTA sodium solution to prevent coagulation. Blood was kept on ice and centrifuged for 3 min at 13,200 rpm in a desktop centrifuge to collect plasma. 25 µL plasma samples were combined with 75 µL internal standard (2 µM warfarin) in acetonitrile in a 96 well plate and centrifuged at 4000 rpm for 20 min at 4 °C. The supernatant (40 µL) was collected and mixed with 2 parts of Milli-Q water and centrifuged again at 4000 rpm for 20 min at 4 °C. Plasma concentration was determined with a partially validated LC/MS-MS assay with multiple reaction monitoring detection (AB Sciex, Framingham, MA). The summary of the LC/MS/MS method is provided below. The assay lower limit of quantification (LLOQ) was 4.57 nM in plasma.

The processed plasma concentration-time data were analyzed using non-compartmental analysis (NCA) in WinNonlin 6.1 with a “plasma model” (200-202, Phoenix, WinNonlin, Pharsight); all standard NCA parameters were estimated via default software settings, using predicted as opposed to observed parameter estimates. The Area Under the Concentration-Time Curve (AUC) was calculated with the linear trapezoidal, linear interpolation rule using mean concentrations and nominal times. The standard error of the AUC to the last time point (AUClast) was generated using the sparse sampling option. The terminal elimination rate (Lambda_z) and half-life (HL_Lambda_z) was determined using the default “Best Fit” method. The predicted AUC from the last time point to infinity (AUCINF_pred) was calculated as AUClast plus Clast(pred)/Lambda_z. Clearance (CL or CL/F) was calculated as Dose/AUCINF_pred. If ≥2/3rds of the observed concentrations were below LLOQ, the mean concentration was treated missing. The results of the non-compartmental analysis for both routes are shown in Table 3.

**LC/MS/MS methods**

| Instrument | AB Sciex 6500 coupled to Waters Acquity UPLC (LC-MS/MS) |
| --- | --- |
| Detection | Positive electrospray ionization multiple reaction monitoring (MRM) mode |
| Column | Acquity BEH C18, 1.7 µm, 2.1*50 mm |
| LC condition | Gradient (99-2 % A) cycle time, 2 min; injection vol: 3 µL; Flow rate: 0.7 mL/min |
| Mobile phase | Acetonitrile (B) –Water (A) gradient with 0.1% formic acid |

| Analyte | MRM Transition (m/z) | MRM_Rt (min) |
| --- | --- | --- |
| Compound 1 | 479.0 > 101.1 | 0.86 |
| Warfarin (IS) | 309.1 > 163.1 | 1.23 |

**Pilot mouse pharmacokinetic study for compounds 4 and 5**

The calibration curve was obtained as follows. The calibration was done using the same biological matrices to ensure similar recovery. Serial dilution (1:2) of compound stock solution (in DMSO) was spiked into 99 volumes of blank mouse plasma (final concentration from 4.5 to 10000 nM). One volume of spiked plasma was combined with three volumes of internal standard (2 µM warfarin in acetonitrile) to precipitate the mouse plasma proteins. All concentrations in the calibration range must be no more than 20% deviation. If there were any points with more than 20% deviation on the high or low end, the calibration range would be truncated. If an outlier occurred in the middle of the curve, the whole curve would be re-done. The signal: noise of the compound peak at the H/LLOQ must be greater than 5. Partial validation is achieved if >75% duplicated calibrations, >2/3rd of total QCs, >50% QC from each level, > meet acceptance interval <20% for LLOQ, 15% for other levels. The samples were tested on the same or the second day.

| Quality control data – Compound **4** | |
| --- | --- |
| QC^a^ (nM) | Accuracy (%) |
| 50 (n=6) | 88.4 |
| 500 (n=6) | 89.6 |
| 5000 (n=4) | 89.1 |

| Quality control data – Compound **5** | |
| --- | --- |
| QC^a^ (nM) | Accuracy (%) |
| 10(n=3) | 112.3 |
| 5000(n=3) | 99.7 |
| 5000 (n=3) | 89 |

**Chemical structure network graph in Figure 2**

Node and edge assignments were made using a custom Pipeline Pilot application. Scaffolds were defined from molecules using the “MurckoAssemblies” option in the Generate Fragments component (exocyclic double bonds and linker double bonds were included, and alpha atoms were treated as R-attachment points). Cores were defined from molecules using the same Pipeline Pilot component, but excluding alpha atoms and linker double bonds, and converting all bonds into single bonds. Five categories of edges were assigned. Molecule nodes were connected to other molecule nodes that shared the same parent scaffold. Edges were also assigned between molecule nodes and their parent scaffold node. Edges were assigned between a parent scaffold node and a child scaffold that contained the parent substructure. Scaffold nodes that could not be assigned a parent were assigned to core nodes. Core nodes were hierarchically clustered using the McQuitty method and the Dixon-Koehler distance metric [3] (Pipeline Pilot ECFP_4 fingerprint). This algorithm produced a connected graph (all nodes had at least one connection). The network was visualized in Cytoscape (v 2.6.3) using the Y-files circular layout method [3].

**References**

1. Di L, Kerns EH, Li SQ, Petusky SL. High throughput microsomal stability assay for insoluble compounds. International Journal of Pharmaceutics. 2006;317(1):54-60.

2. Dixon SL, Koehler RT. The hidden component of size in two-dimensional fragment descriptors: side effects on sampling in bioactive libraries. J Med Chem. 1999;42(15):2887-900. doi: 10.1021/jm980708c. PubMed PMID: 10425098.

3. Shannon P, Markiel A, Ozier O, Baliga NS, Wang JT, Ramage D, et al. Cytoscape: a software environment for integrated models of biomolecular interaction networks. Genome Res. 2003;13(11):2498-504. doi: 10.1101/gr.1239303. PubMed PMID: 14597658; PubMed Central PMCID: PMCPMC403769.
